# TG/HDL-C Ratio for Predicting Insulin Resistance in Obese Children from Beijing, China

**DOI:** 10.2174/0118715303245154231023104618

**Published:** 2023-11-01

**Authors:** Tian Zhang, Fangfang Duan, Yi Qian, Jin Zhang, Huihui Sun, Naijun Wan

**Affiliations:** 1 Department of Paediatric Internal Medicine, Beijing Jishuitan Hospital, Capital Medical University, Beijing, 100035, China;; 2 Department of Clinical Epidemiology Research, Beijing Jishuitan Hospital, Capital Medical University, Beijing, 100035, China

**Keywords:** Obesity, children, TG/HDL-C ratio, insulin resistance, HOMA-IR, triglycerides, high-density lipoproteins

## Abstract

**Background:**

International studies have found that the blood triglycerides to high-density lipoproteins (TG/HDL-C) ratio predicted insulin resistance in children with overweight and obesity. However, there is a lack of such reports on children from China.

**Objective:**

The objective of this study is to explore the ability of the TG/HDL-C ratio as a blood biomarker for insulin resistance (IR) in obese children in Beijing.

**Methods:**

We evaluated 262 children with obesity from our paediatric outpatient clinic in a cross-sectional study. Detailed medical histories of all children were ascertained, as were clinical examination and laboratory test results, including blood lipids, fasting glucose, insulin, and glycated haemoglobin. We divided them into age groups of 6-9 and 10-13.5 years and then into IR and non-IR groups based on the homeostatic model assessment for IR (HOMA-IR). Analysis was accomplished with SPSS software (version 22.0).

**Results:**

The TG/HDL-C ratio was higher in children with IR in the 6-9 and 10-13.5-year age groups (*p* < 0.001). Univariate and multivariate analyses displayed that the TG/HDL-C ratio and HOMA-IR were correlated in the 6-9 and 10-13.5-year-old groups (*p* < 0.05). In the 6-9-year-old group, IR identified by a TG/HDL-C ratio ≥ 0.645 had a sensitivity, specificity, and an area under the curve (AUC) of 79.1%, 60.9%, and 0.734, respectively. In the 10–13.5-year-old group, IR identified by a TG/HDL-C ratio ≥ 0.725 had a sensitivity, specificity, and an AUC of 79.4%, 62.9%, and 0.724, respectively.

**Conclusion:**

We showed the application of the TG/HDL-C ratio to predict insulin resistance in obese children in Beijing with different diagnostic thresholds based on age (6-9-year-old group with TG/HDL-C ≥ 0.645; 10–13.5-year-old group with TG/HDL-C ≥ 0.725), which were lower compared with the diagnostic threshold for insulin resistance in children reported in other countries.

## 1 INTRODUCTION

As the Chinese economy develops, the prevalence of obese children and adolescents increases annually [1, 2]. Studies have found that obesity is an important risk factor for diabetes and insulin resistance (IR) [3]. The incidence rate of type 2 diabetes in children and adolescents aged 6-18 years in Beijing is 0.6/1,000 [4]. Predicting IR is a major indicator for the prevention and early intervention of type 2 diabetes [5]. Currently, the homeostatic model assessment for IR (HOMA-IR) is utilised in clinics to evaluate children and adolescents [5, 6]. However, the cost of blood insulin testing is relatively high compared with blood triglycerides (TGs) and high-density lipoproteins (HDLs). In addition, blood insulin testing is not yet widespread at primary hospitals. In patients with obesity, TGs and HDLs are tested at primary hospitals and are easily incorporated into routine physical examinations at a relatively low cost [7]. Some international studies have described that the ratio of TGs to HDLs in obese children has high specificity and sensitivity as a predictor of IR [7, 8]. However, the cut-off values of the TG/HDL ratio vary among different ethnic groups [9]. The use of this ratio as a predictor of IR in adults has also been reported in China [10]. However, there is a lack of such reports on children from China. Here, we explored the usefulness of the TG/HDL-C ratio in forecasting IR in obese children in Beijing.

## 2 MATERIALS AND METHODS

### 2.1 Enrolled Children

When the body mass index (BMI) of the child is equal to or higher than the standard value for obesity in children of the same age and sex, the child is diagnosed with obesity. A total of 262 children with simple obesity who attended our paediatric outpatient clinic between August 2018 and May 2022 were enrolled and tested at our hospital. All children were informed about the study aims, and the parents gave their written consent. We included children with obesity who were screened according to the 2018 Chinese criteria of “Screening for Overweight and Obesity in School-Aged Children and Adolescents” [11]. The exclusion criteria were as follows concomitant obesity-related diseases such as diabetes and/or cardiovascular diseases; other concomitant major diseases such as liver and kidney diseases or psychiatric diseases; and hereditary obesity. The study protocol was endorsed by the Institutional Review Board of Beijing Jishuitan Hospital.

### 2.2 Data Processing

#### 2.2.1 Patient Characteristics

Medical history, clinical examination, and questionnaire responses were recorded using standardised spreadsheets. Height and weight were assessed on the same specialised column scale with an accuracy of 0.1 cm and 0.1 kg, respectively. The BMI was computed as follows: weight (kg) / height (m)^2^. Height, weight, and BMI were standardised based on the relevant standard deviations of Chinese children of the same age and sex [12, 13] and represented by the median.

#### 2.2.2 Laboratory Tests

All children observed fasting, which included abstinence from food and water intake from 9 p.m. the night before until the time of blood collection, totalling 10 hours of fasting. Venous blood sampling was performed in the morning. Fasting glucose, glycated haemoglobin, lipids, and insulin levels were gauged. Fasting blood glucose and blood lipids were tested using the Hitachi 7600 automated biochemistry analyser. Glycated haemoglobin was measured using the Tosoh G8 instrument and its reagents. Insulin was measured using the Roche E602 instrument and its reagents. HOMA-IR was computed as follows: fasting glucose (mmol/l)* fasting insulin (IU/ml) / 22.5. HOMA-IR ≥ 4.0 was classified as IR, and HOMA-IR < 4.0 points were classified as insulin sensitivity [5, 8].

### 2.3 Data Analysis

The analyses were computed using the SPSS software (version 22.0). Categorical variables are detailed with proportions and percentages. The chi-square test (X^2^) or Fisher’s exact test was utilised for comparing categorical groups. The Shapiro-Wilk test was employed to test the normality of quantitative variables. Normally distributed quantitative variables are expressed using mean ± standard deviation. The independent *t*-test was utilised for comparing the groups. Non-normally distributed quantitative variables are expressed using the interquartile range and median. The Mann-Whitney U test was employed for comparisons. Spearman’s correlation analysis was utilised for investigating correlations among the TG/HDL-C ratio and quantitative variables. Logistic regression analysis was adopted to characterise the relationship between the TG/HDL-C ratio and IR. We estimated several parameters related to the TG/HDL-C ratio accuracy in forecasting IR. Statistical discrepancies with *p* < 0.05 were considered significant. The receiver operating characteristic (ROC) curve was used to determine the optimal cut-off of the TG / HDL ratio for judging insulin resistance, and to estimate the sensitivity, specificity, positive predictive value, negative predictive value and consensus rate of TG / HDL when predicting IR at this cut-off.

## 3 RESULTS

### 3.1 Baseline Characteristics

In total, 262 obese children, including 148 boys and 114 girls ranging from 6 to 13.5 years old (mean age: 9.39 ± 1.82 years), were enrolled. Children were classified into the IR group (n = 135, 51.5%) and the non-IR group (n = 127,48.5%) based on HOMA-IR values. Table **1** shows the baseline features of 262 obese children according to age. There were 159 children aged 6-9 years and 103 children aged 10-13.5 years. The prevalence of IR in children aged 6-9 years was 42.1% (67/159), while it was 66.0% (68/103) in children aged 10-13.5 years. There was no statistically significant difference in sex distribution between the IR and non-IR groups in children aged 6-9 years. However, there were statistically significant differences in age, height standard deviation (height SDS), standard deviation of body weight (weight SDS), BMI z-score (BMI z-score), and waist-to-hip ratio (WHR). There was no statistically significant difference in age, sex distribution, height SDS or WHR between the IR and non-IR groups of children aged 10-13.5 years. However, there was a statistically significant difference in weight SDS, and BMI z-score.

### 3.2 Analysis of Baseline Characteristics Metabolic Characteristics According to Age

Table **2** shows metabolic characteristics at 6-9 and 10-13.5 years. The blood levels of fasting glucose, insulin, glycated haemoglobin, triglycerides, uric acid, TG/HDL-C ratio, and low-density lipoprotein of children in the 6-9-year-old group with IR were significantly higher than those in the non-IR group, while high-density lipoprotein was significantly lower than that in the non-IR group. In the 10-13.5-year-old group, statistically significant differences existed in blood levels of fasting glucose, insulin, triglycerides, and TG/HDL-C ratio between the IR and non-IR groups.

### 3.3 Interactions between TG/HDL-C Ratio and HOMA-IR

Table **3** shows age, weight SDS, height SDS, BMI z-score, insulin, blood glucose, glycated haemoglobin, TGs, uric acid, LDLs, and TG/HDL-C levels positively related to HOMA-IR values in the 6-9-year-old group. In the 10-13.5-year-old group, weight SDS, BMI z-score, WHR, insulin, blood glucose, TGs, and TG/HDL-C ratio were still positively related to HOMA-IR values. All variables found to be correlated with IR were imported into the logistic regression output. Table **4** shows that after adjusting for other factors such as insulin and fasting glucose levels, the statistically significant correlation persisted in the 6-9-year-old (odds ratio [OR]: 3.620, 95% confidence interval [CI]: 1.418,9.241, *p* = 0.007) and 10-13.5-year-old groups (OR: 6.060, 95% CI: 1.842,19.933, *p* = 0.003).

### 3.4 ROC Curve

**Fig. (1):**
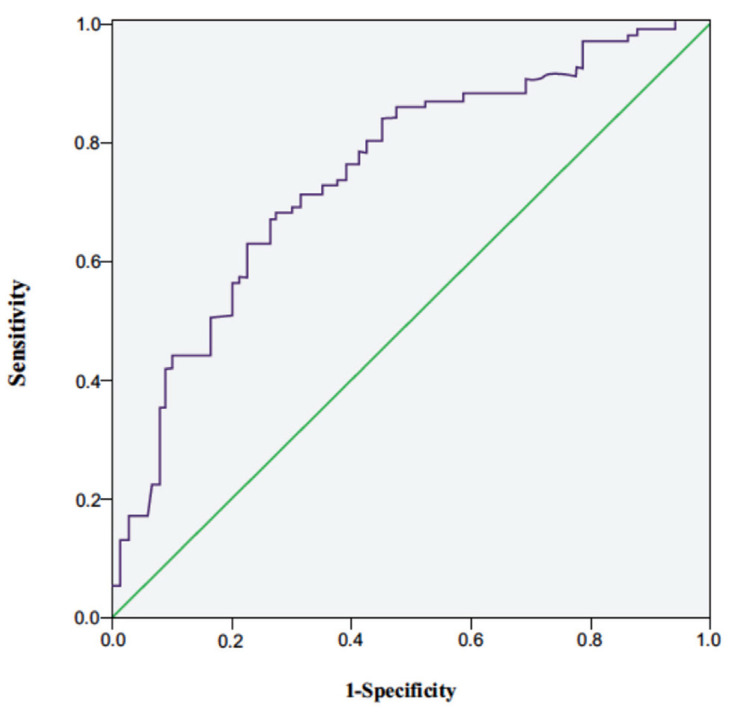
ROC curve utilising the TG/HDL-C ratio to forecast IR at 6-9 years. **Abbreviations:** ROC, receiver operating characteristic; TG/HDL-C, triglycerides to high-density lipoproteins ratio.

**Fig. (2):**
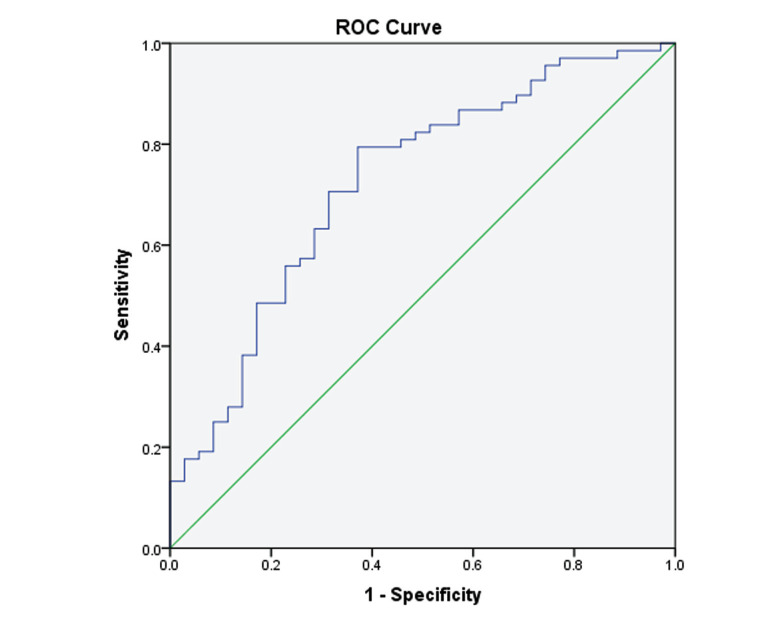
ROC curve utilising the TG/HDL-C ratio to forecast IR at 10-13.5 years. **Abbreviations:** ROC, receiver operating characteristic; TG/HDL-C, triglycerides to high-density lipoproteins ratio.

ROC curves can be used to analyse the clinical value of TG/HDL-C in diagnosing insulin resistance in obese children and to determine the optimal diagnostic threshold when the Jordan index is maximum. In the 6-9-year-old group, the Youden index of the receiver operating characteristic (ROC) curve was highest at a ratio of 0.645. With this cut-off value, evaluating IR was characterised by 79.1% sensitivity and 60.9% specificity. The area under the ROC curve (AUC) was 0.734 (Fig. **1**, Table **5**). In the 10-13.5-year-old group, the Youden index of the ROC curve was highest at a ratio of 0.725. With this cut-off value, evaluating IR was characterised by 79.4% sensitivity and 62.9% specificity. The AUC was 0.724 (Fig. **2**, Table **6**).

## 4 DISCUSSION

Patients with diabetes and IR often present clinically with elevated blood TGs and LDLs levels and reduced blood HDLs levels [14]. Studies have confirmed that high triglycerides and low HDL levels are positively associated with diabetes and IR [15]. Therefore, a high TG/HDL-C ratio may suggest the existence of IR.

The hyperinsulinemic hyperglycaemic clamp technique is the gold standard for diagnosing IR, but the method is complex, and its clinical application is limited [16]. Nowadays, HOMA-IR is widely used in clinical practice to assess the IR status of children and adolescents [5, 6]. Therefore, this study used HOMA-IR to divide obese children into IR and non-IR groups and analysed the correlation between TG/HDL-C and HOMA-IR to further evaluate the clinical value of TG/HDL-C in diagnosing IR in obese children in Beijing.

We showed that the incidence of insulin resistance was 42.1% in the 6-9-year-old group, while it was 66.0% in the 10-13.5-year-old group, indicating that age is an important factor in the development of insulin resistance in obese children. This is consistent with Hirschler *et al.*’s results, whose study on the application of the TG/HDL ratio to predict insulin resistance in Argentinean indigenous children analysed IR in children of different age groups, showing a higher degree of IR in children of older age [17]. Therefore, we evaluated the value of using TG/HDL-C to predict insulin resistance in obese children of different ages. In 6-9-year-old children, we found that age, BMI z-score, height SDS, weight SDS, WHR, fasting glucose, glycated haemoglobin, insulin, TGs, LDLs, and uric acid were higher in children with IR compared with non-IR children. Conversely, HDL-C levels were lower in IR children compared with non-IR children. In 10-13.5-year-old children, BMI z-score, weight SDS, BMI z-score, WHR, fasting glucose, insulin, and TGs were still higher in IR children compared with non-IR children. This result is consistent with the metabolic characteristics of patients with IR. The mechanism of IR is currently studied as a biological response to impaired insulin stimulation in the liver, muscle, and adipose tissue, resulting in hyperinsulinemia. Moreover, patients with IR can develop hyperglycaemia, hypertension, dyslipidaemia, visceral obesity, hyperuricaemia, elevated inflammatory markers, endothelial dysfunction, and a hypercoagulable state, thus leading to diabetes, metabolic syndrome, and non-alcoholic fatty liver disease [18]. Studies have also demonstrated that WC, WHR, and waist-to-height ratio are measures of central obesity [19], whereas visceral fat is independently related to IR [20]. A retrospective study of 15,198 Chinese adults reported a relationship between the TG/HDL-C ratio and hyperuricemia [21]. A study by Behiry *et al*. [8] also confirmed that the TGs, waist circumference, BMI z-score, and body fat percentage of children in the IR group were significantly higher than those in the non-IR group. Therefore, when determining the relationship between TG/HDL-C ratio and IR, we explored whether there was still a correlation among children of different age groups under the influence of the above factors.

We showed that the TG/HDL-C ratio remained related to HOMA-IR after excluding the confounding effects of age, sex, weight, BMI z-score, WHR, uric acid, LDLs, and glycated haemoglobin in the 6-9-year-old and 10-13.5-year-old groups. The diagnostic ROC curve was characterised by the largest AUC at a predictive cut-off value of 0.645 in the 6-9-year-old group and 0.725 in the 10-13.5-year-old group. Gasevic *et al.* evaluated the TG/HDL-C ratio to predict IR in different populations, revealing that TG/HDL-C prediction cut-off values differed among ethnicities, with an optimal cut-off value of 1.1 for Chinese and 0.9, 1.2, and 1.8 for Aboriginals, Europeans, and South Asians, respectively [9]. International research demonstrated that the TG/HDL-C ratio was used to predict IR in overweight and obese children [7-9, 22], but there is a lack of such reports on children from China. The results of this study are consistent with the ROC curve boundaries (0.65-0.75) utilising the TG/HDL-C ratio to forecast IR in Chinese adults [8], suggesting its ability to provide a reference value for the prediction of IR in children with obesity in Beijing.

There are some limitations and drawbacks to this study. This was a cross-sectional study enrolling only children with obesity residing in the urban part of Beijing (aged 6-13.5 years). At present, there is relatively little data available, and the analysis of the data in this study did not achieve a classification discussion based on sex and pubertal stage. Therefore, the sample size should be expanded in a multicentre, longitudinal study to further determine a rational threshold for IR in children with obesity in China.

## CONCLUSION

The TG/HDL-C ratio was an easy and cheap test for predicting IR in obese children. Age is an important factor in the development of insulin resistance in obese children; therefore, we demonstrated the application of the TG/HDL-C ratio to predict insulin resistance in obese children in Beijing with different diagnostic thresholds based on age (6-9-year-old group with TG/HDL-C≥0.645; 10-13.5-year-old group with TG/HDL-C ≥0.725), which were lower compared with the diagnostic threshold for insulin resistance in children reported in other countries.

## Figures and Tables

**Table 1:** Baseline features of 262 obese children.

**Variable**	**IR Group**	**Non-IR Group**	**T or χ^2^**	***p***
Age (Years) ± s	9.93 ± 1.71	8.81 ± 1.77	-5.233	< 0.001
Sex (m:f)	82:53	66:61	2.049	0.152
**6-9 Years**				
Age (Years) ± s	8.53 ± 1.01	7.96 ± 1.17	-3.287	0.001
Sex (m:f)	38:29	42:50	1.898	0.168
Weight SDS M (P25, P75)	4.39 (3.36, 5.43)	3.03 (2.34, 4.76)	-3.485	0.001
Height SDS M (P25, P75)	2.18 (1.30, 2.68)	1.31 (0.66, 2.57)	-2.240	0.026
BMI z-score M (P25, P75)	3.15 (2.57, 3.59)	2.60 (2.29, 3.08)	-3.811	< 0.001
WHR M (P25, P75)	0.84 (0.79, 0.87)	0.78 (0.75, 0.83)	-3.175	0.002
**10-13.5 Years**				
Age (Years) ± s	11.32 ± 0.95	11.05 ± 0.88	-1.388	0.168
Sex (m:f)	44:24	24:11	0.154	0.695
Weight SDS M (P25, P75)	3.65 (2.36, 4.35)	2.30 (1.52, 3.42)	-2765	0.007
Height SDS M (P25, P75)	1.36 (0.55, 2.18)	1.15 (0.28, 1.89)	-1.852	0.067
BMI z-score M (P25, P75)	2.56 (2.25, 3.06)	2.27 (1.84, 2.71)	-2.444	0.016
WHR M (P25, P75)	0.88 (0.84, 0.93)	0.85 (0.81, 0.89)	-1.719	0.089

**Abbreviations:** BMI z-score, body mass index z-score; IR, insulin resistance; WHR, waist-to-hip ratio.

**Table 2:** Biochemical indicators in IR groups according to age.

**Variable**	**IR Group**	**Non-IR Group**	**T or U**	***p***
**6-9 Years**				
Blood glucose (mmol/l) M (P25,P75)	5.10 (4.80,5.40)	4.90 (4.70,5.10)	2,172.50	0.001
Insulin (IU/ml) M (P25,P75)	27.30 (21.30,36.00)	11.90 (9.00,14.80)	32.50	< 0.001
Glycated haemoglobin (%) M (P25,P75)	5.50 (5.30,5.60)	5.40 (5.20,5.60)	2,436.00	0.023
TGs (mmol/l) M (P25,P75)	1.32 (0.97,1.72)	0.83 (0.62,1.22)	1,614.00	< 0.001
HDLs (mmol/l) M (P25,P75)	1.34 (1.16,1.49)	1.45 (1.32,1.62)	2,238.50	0.003
Total cholesterol (mmol/l) M (P25, P75)	4.53 (4.22,5.13)	4.38 (4.01,5.05)	2,584.00	0.082
LDLs (mmol/l) M (P25, P75)	2.74 (2.47,3.32)	2.51 (2.09,2.91)	2,282.50	0.005
TG/HDL-C M (P25, P75)	1.03 (0.67,1.30)	0.54 (0.39,0.85)	1,639.00	< 0.001
Uric acid (μmol/l) M (P25, P75)	369.00 (317.00,405.00)	332.00 (290.00,367.75)	2,262.50	0.004
**10-13.5 Years**				
Blood glucose (mmol/l) M (P25, P75)	5.10 (4.82,5.38)	4.90 (4.70,5.10)	833.50	0.013
Insulin (IU/ml) M (P25, P75)	31.45 (25.45,43.8)	12.90 (10.60,15.20)	8.00	< 0.001
Glycated haemoglobin (%) M (P25, P75)	5.50 (5.40,5.70)	5.50 (5.30,5.50)	906.00	0.058
TGs (mmol/l) M (P25, P75)	1.35 (1.02,1.80)	0.90 (0.70,1.40)	675.50	< 0.001
HDLs (mmol/l) M (P25, P75)	1.28 (1.13,1.44)	1.33 (1.25,1.57)	911.00	0.052
Total cholesterol (mmol/l) M (P25, P75)	4.41 (3.87,4.84)	4.14 (3.67,4.92)	1,107.50	0.566
LDLs (mmol/l) M (P25, P75)	2.48 (2.09,2.96)	2.45 (1.85,2.85)	1,096.00	0.513
TG/HDL-C M (P25, P75)	1.06 (0.78,1.06)	0.67 (0.47,0.99)	656.00	< 0.001
Uric acid (μmol/l) M (P25, P75)	404.00 (332.75,460.25)	381.00 (326.00,418.00)	985.50	0.154

**Abbreviations:** HDLs, high-density lipoproteins; IR: insulin resistance; LDLs, low-density lipoproteins; TGs, triglycerides.

**Table 3:** Interactions between various indicators and HOMA-IR.

**Variable**	**6-9 Years**		**10-13.5 Years**	
	**Correlation Coefficient**	***p***	**Correlation Coefficient**	***p***
Age	0.242	0.002	0.138	0.165
Sex	0.109	0.170	-0.039	0.698
Weight SDS	0.274	< 0.001	0.275	0.005
Height SDS	0.193	0.015	0.121	0.233
BMI z-score	0.295	< 0.001	0.242	0.014
WHR	0.265	0.001	0.211	0.034
Blood glucose	0.253	0.001	0.247	0.012
Insulin	0.847	< 0.001	0.815	< 0.001
Total cholesterol	0.138	0.082	0.057	0.568
TG/HDL-C	0.400	< 0.001	0.368	< 0.001
Glycated haemoglobin	0.181	0.023	0.189	0.057
TGs	0.407	< 0.001	0.355	< 0.001
HDLs	-0.234	0.003	-0.192	0.052
LDLs	0.222	0.005	0.065	0.515
Uric acid	0.227	0.004	0.141	0.155

**Abbreviations:** Bmi Z-Score, Body Mass Index Z-Score; Hdls, High-Density Lipoproteins; Ldls, Low-Density Lipoproteins; Tgs, Triglycerides; Whr, Waist-To-Hip Ratio.

**Table 4:** Multivariate analysis.

**Variable**	**6-9 Years**			**10-13.5 Years**		
	***β***	***p***	**OR (95% CI)**	***β***	***p***	**OR (95% CI)**
TG/HDL-C	1.287	0.007	3.620 (1.418,9.241)	1.802	0.003	6.060 (1.842,19.933)
Age	0.558	0.015	1.747 (1.113,2.742)	0.321	0.298	1.379 (0.753,2.523)
BMI z-score	0.963	0.006	2.621 (1.317,5.215)	0.729	0.124	2.074 (0.818,5.257)
WHR	1.115	0.767	3.049 (0.002,4866.475)	1.911	0.568	6.759 (0.010,4735.518)
Uric acid	0.001	0.921	1.000 (0.994-1.007)	0.001	0.983	1.000 (0.993-1.007)
Glycated haemoglobin	-0.372	0.497	0.690 (0.236,2.014)	1.400	0.163	4.055 (0.566,29.059)
LDLs	0.355	0.278	1.426 (0.751,2.711)	-0.071	0.850	0.932 (0.448,1.936)

**Abbreviations:** Bmi Z-Score, Body Mass Index Z-Score; Ci, Confidence Interval; Df, Degrees Of Freedom; Hdl, High-Density Lipoproteins; Ldls, Low-Density Lipoproteins; Whr, Waist-To-Hip Ratio.

**Table 5:** ROC curve utilising the TG/HDL-C ratio to forecast IR at 6-9 years.

**-**	**Sens**	**Spec**	**PPV**	**NPV**	**AUC**	**Accuracy**	**95% CI**	***p***
TG/HDL-C ≥ 0.645	0.791	0.609	0.669	0.744	0.734	0.700	0.656-0.813	< 0.001

**Abbreviations:** AUC, area under the receiver operating characteristic curve; CI, confidence interval; NPV, negative predictive value; PPV, positive predictive value.

**Table 6:** ROC curve utilising the TG/HDL-C ratio to forecast IR at 10-13.5 years.

**-**	**Sens**	**Spec**	**PPV**	**NPV**	**AUC**	**Accuracy**	**95%CI**	***p***
TG/HDL-C ≥ 0.725	0.794	0.629	0.681	0.753	0.724	0.712	0.619-0.829	< 0.001

**Abbreviations:** AUC, area under the receiver operating characteristic curve; CI, confidence interval; NPV, negative predictive value; PPV, positive predictive value.

## Data Availability

All data generated or analysed during this study are included in this published article.

## LIST OF ABBREVIATIONS

BMI z-score: Body Mass Index z-score
HDL-C: High-density Lipoprotein Cholesterol
IR: Insulin Resistance
LDL-C: Low-density Lipoprotein Cholesterol
TG: Triglyceride
WC: Waist Circumference
WHR: Waist-to-hip Ratio

## STANDARDS OF REPORTING

STROBE guidelines were followed in the study.
